# Whole-Genome Sequences of the Archetypal K1 Escherichia coli Neonatal Isolate RS218 and Contemporary Neonatal Bacteremia Clinical Isolates SCB11, SCB12, and SCB15

**DOI:** 10.1128/genomeA.01598-14

**Published:** 2015-02-26

**Authors:** Michael W. Day, Lydgia A. Jackson, Darrin R. Akins, David W. Dyer, Susana Chavez-Bueno

**Affiliations:** a Department of Microbiology and Immunology, University of Oklahoma Health Sciences Center, Oklahoma City, Oklahoma, USA; b Department of Pediatrics, University of Oklahoma Health Sciences Center, Oklahoma City, Oklahoma, USA

## Abstract

Neonatal bacteremia Escherichia coli strains commonly belong to the K1 capsular type. Their ability to cause invasive neonatal disease appears to be determined by other virulence factors that have yet to be identified. We report here the genome sequences of four E. coli neonatal bacteremia isolates, including that of the archetypal strain RS218.

## GENOME ANNOUNCEMENT

Escherichia coli is the most common cause of Gram-negative bacteremia in newborns and young infants and is associated with a mortality rate approaching 40% (1, 2). High-degree E. coli bacteremia can also result in meningitis, which commonly leads to long-term or permanent neurological sequelae (3). The majority of neonatal E. coli isolates causing bacteremia and meningitis express the K1 capsular antigen, which confers serum resistance and protects against phagocytic killing (4–6). RS218 is a well-characterized K1-positive neonatal E. coli meningitic isolate recovered in 1974 from the cerebrospinal fluid of a newborn (7, 8). Several RS218 virulence factors, such as OmpA, Ibe, and CNF1, are integral to bacterial passage across the blood-brain barrier (9). The identification and characterization of additional bacterial factors in the pathogenesis of RS218 and other invasive neonatal E. coli strains remain important areas of research. Here, we present the whole-genome sequences of RS218 and three contemporary E. coli blood culture isolates, SCB11, SCB12, and SCB15, which were identified in bacteremic newborns hospitalized at our institution in 2007 (10).

We performed whole-genome sequencing of the four isolates on an Illumina MiSeq using a 250-bp paired-end library. Assembly was performed *de novo* with the A5 assembly pipeline. The sequencing characteristics for each strain are summarized in Table 1. The annotation of the genomes was performed using the NCBI Prokaryotic Genomes Annotation Pipeline. The *adk*, *fumC*, *gyrB*, *icd*, *mdh*, *purA*, and *recA* genes in RS218 were consistent with its multilocus sequence type 95 (ST95). An examination of this same gene set in SCB11, SCB12, and SCB15 indicated they corresponded to ST141, ST95, and ST501, respectively (11). RS218, SCB11, and SCB12 belong to phylogroup B2, whereas SCB15 is in phylogroup D. All four isolates carry the *kpsM* II group 2 capsule genes and express the K1 capsular antigen, as assessed by agglutination testing. Other known extraintestinal pathogenic E. coli (ExPEC) virulence genes, including *cnf1*, *fyuA*, *hek*, *hlyC*, *ibeA*, *iroN*, *papGII*, and *sfa* (12), were observed in the RS218, SCB11, and SCB12 genomes, but only *fyuA* and *sfa* were identified in SCB15. As expected, the recently identified plasmid pRS218 was present in strain RS218 (13). SCB12 was found to contain 90% of the pRS218 published sequence. SCB11 and SCB15, in contrast, do not contain pRS218. Future comparisons between these contemporary clinical isolates and the archetypal RS218 strain will yield valuable insight into the molecular pathways exploited by different E. coli isolates causing neonatal septicemia and meningitis.

**TABLE 1:** Genome sequencing statistics and accession numbers for neonatal invasive E. coli clinical isolates

Strain name	Total contig length (bp)	No. of contigs	G+C content (%)	*N*_50_ (bp)	GenBank accession no.	Accession no. version
RS218	5,173,885	68	50.6	277,884	JWZW00000000	JWZW01000000
SCB11	5,105,498	74	50.4	274,108	JSYT00000000	JSYT01000000
SCB12	5,478,295	96	50.5	144,019	JMQO00000000	JMQO01000000
SCB15	4,920,323	141	50.5	174,737	JSYU00000000	JSYU01000000

### Nucleotide sequence accession numbers.

The nucleotide sequences have been deposited at GenBank under the accession numbers listed in Table 1.
